# How self-determination of scholars outclasses shrinking public research lab budgets, supporting scientific production: a case study and R&D management implications

**DOI:** 10.1016/j.heliyon.2021.e05998

**Published:** 2021-01-28

**Authors:** Mario Pagliaro, Mario Coccia

**Affiliations:** a Istituto per lo Studio dei Materiali Nanostrutturati, CNR, via U. La Malfa 153, 90146 Palermo, Italy; b Istituto di Ricerca sulla Crescita Economica Sostenibile, CNR, via Real Collegio 30 (Collegio Carlo Alberto), 10024 Moncalieri (TO), Italy

**Keywords:** Public research lab, Organizational behavior, Self-determination, R&D management, Economic crisis, Public funds, Budgetary policies, Scientific productivity, Reputation, Scholarly production, Research policy

## Abstract

The main purpose of this study is to analyze how reorganization of public research organizations and shrinking public research lab budgets affect the evolution of research activity and scientific productivity. This study focuses on a case study of National Research Council of Italy (CNR), one of largest European public research organizations, to identify evolution of research activities and dynamics of scientific production from 2000 to 2019. The statistical analysis suggests that, in line with the origin of the CNR, scientific research focuses mainly on research fields of natural sciences, such as chemical, life and physical sciences, with an increasing role of scientific research in energy, engineering and mathematics. The study also shows a high intensity of collaboration of the CNR within international research networks. A key finding of this study is to show, for the first time, that although uncertain reforms and reductions of public funds, scientific productivity is growing. This novel result can be explained with self-determination of scholars as a vital determinant supporting scientific production that outclasses shrinking public research lab budgets and organizational deficiencies. The management and research policy implications of this study can be generalized to support an efficient organizational and managerial behavior, and higher scientific productivity of public research institutes in contexts of reduced public funding and market turbulence.

## 1 Introduction

This study has two goals. The first is to analyze the organizational behavior of public research organizations in performing scientific research in the presence of reforms and shrinking public research lab budgets. The second is to explain the typology and dynamics of scientific production in these contexts for best practices of Research and Development (R&D) management to sustain, whenever possible, an efficient organizational behavior of public research organizations in turbulent environments. These topics extend previous studies on organizational and managerial behavior of Public Research Organizations (PROs) and scientific production in rapidly changing economies and research markets [1,2,3,4,5].

To achieve the study's goals, we analyze the “strategic change” [6] of Italy's largest public research organization, the National Research Council of Italy (CNR) from 2000 to 2019. CNR in Italy is a PRO similar to other large European public research institutions, such as Centre National de la Recherche Scientifique (CNRS) in France, Consejo Superior de Investigaciones Científicas (CSIC) in Spain, etc. [7,8,9]. The CNR carries out, promotes, and spreads scientific research activities aimed to foster the scientific, technologic, economic, and social progress of Italy. Its scientific activities are focused on: biomedical sciences, physics and technology of the matter, science of earth system and technologies for environment, chemistry and material technologies, engineering, information and communication technologies, technologies for energy and transportation, biological and food sciences, socio-economic sciences, human sciences and cultural heritage [10].

The CNR has an organization based on a wide network of institutes and decentralized units in almost all Italian regions. This network organization facilitates a diffusion of scientific competences into the economic system and fosters collaborations with local institutions and businesses. The operation of CNR is driven by a three-year activity plan (updated yearly) that sets general guidelines, objectives, priorities, and resources consistent with the National Research Program of Italy and with the research programs of the European Union. The three-year plan specifies the financial requirements (*i.e.*, the budget), spells out the implicit human resources requirements and personnel recruitment needs in different scientific fields, schedules projects, and carries out the planned scientific activities. The Italian Ministry of Research reviews and approves the plan. Next section explains different reforms of this large PRO in Europe to create a background for the statistical analysis of the typology and evolution of scientific activity over time.

## 2 Theoretical background

The organization of the Italian CNR has been affected by policy changes carried out in accordance with a shift in governments of Italy and corporate governance of this public scientific institution [11]. The first reform of the CNR in 1999 was inspired by the idea to gain efficiency through the consolidation -- and associated larger sizes -- of research institutes. In particular, the purpose of the first reform was to increase the size of institutes, based on the equation that “large labs = efficient labs” for achieving lower total costs and scale economies in research activity, emulating the large institutes of the Max-Planck-Gesellschaft in Germany. This change in research policy was guided by a narrow administrative stance. Although today there are about 100 consolidated institutes, these new institutes are often composed of several decentralized units (about 330 in 2020) located far from respective headquarters. Such consolidation has generated in some cases increased costs of coordination of decentralized units [12], which in its turn spawns a bureaucratization in organization [13,14]. Moreover, literature indicates that smaller rather than larger sized laboratories can be more productive [15,16,17,18].

While this reform was still underway, a new government in 2003 decided to launch a restructuring of the CNR based on project management principles with the explicit aim to transform the CNR into an entrepreneurial body operating at the service of firms and supplying technological services to small- and medium-sized enterprises and other institutional subjects in Italy [19,20,21]. This second reform has caused further coordination problems among the research units due to the proliferation of channels of communication that creates informational log-jams and conflicts of competencies between some directors of institutes and of departments [12,17,18]. Because of this new organization, many institutes of the CNR are focusing on technological services and applied activities rather than on basic research [22]. In addition, in the past, required financial resources came mainly from Italian governments, but now the CNR receives quite large amounts of funds from private industry and the European Union: in fact, about 40 percent of its total financial report (of 900,000,000 EUR) is third-party financed [16,17,18,19]. These funds are secured through consultancy studies and technological services for external clients, from private businesses, as well as through research grants from the European Union and/or other international bodies. In particular, technological services of the institutes cover manifold activities, such as: analyses and technical tests (chemical and physical); technological services (homologation, calibration, nuclear magnetic resonance, etc.); quality services (accreditation, certification, quality control, etc.); environmental services (water monitoring, pollutant emission control, etc.); information technology services (data elaboration, supply of databases and data, etc.); health services; other research activities based on contracts with firms and public institutions [16]. Like the CNR, other European research institutions have opted for a project-based organization. Mangematin and co-workers in 2006 analyzed [20] the French National Laboratory of Advanced Technologies (NLAT), which decided in 1998 to change its organization from a team-based to a project-based form [21,23].

Looking for explanations and motives of this organizational change, scholars identify the following elements: low project core staffing levels that stimulate the circulation of engineers and researchers among projects blurring project boundaries; implementing and managing thematic projects, which build on specific competencies developed in dedicated projects; and encouraging “bricolage” to hybridize project management with traditional hierarchical management practices. Most NLAT engineers and technicians are not allocated to a specific project full-time, but are supposed to move from one project to another.

This circulation of individuals communalizes project management practices within organization [21,23]. However, such practices tend to destroy the tacit knowledge of scholars linked to projects, and if changes are too frequent, the realization of projects can be slowed and learning processes dissolved. While project management tools have been designed to manage specific projects, the fact that they are not adapted to the management of thematic projects (as well as the fact that project management tools are often not supported by top management) explains why projects are held up frequently [20]. Project management ambiguities regarding the definition of the project and the responsibilities of project leader, etc., induce risks [24]. Frequent delays and inadequacy of project leaders’ answers to managerial problems appear to have produced in some cases a management crisis [20,25]. This literature can clarify the topics of this study on the on-going scientific activities of the CNR.

In this context, economic recession and other crises in Europe are generating ever more shrinking of public research lab budgets [7,8,9]. One of the problems is to analyze and explain typologies of scientific activities and dynamics of scientific production in a period of public reforms and reduction of budgets over time. To the best of our knowledge, no study on the evolution of scientific research of a specific large PRO in problematic contexts, such as Italy, over a long run of twenty years has been performed so far. This study can provide original findings to explain and, whenever possible, generalize, how organizational and managerial behavior of PROs affects the type of research and scientific performance of scholars in different research fields and into the organization as a whole.

## 3 Methodology

The study is based on a case study research [26,27] focused on a large PRO given by Italy's National Research Council (in short, CNR). The study design applies a narrative approach and descriptive statistics that endeavor to explain the evolution of scientific activity and scientific productivity from 2000 to 2019 during a period of reforms and shrinking public research lab budgets. Following Ansari and co-workers [28], a narrative approach is based on a range of data sources. We made use of data and information on CNR's website, including product information, annual reports and published accounts. The source of scientific production was Scopus [29], an abstract and citation database covering more than 36,377 titles from approximately 12,000 publishers, of which more than 34,340 are peer-reviewed journals in top-level subject fields (life sciences, social sciences, physical sciences and health sciences). In addition, data of research personnel (researchers and technicians), and data of financial statements are from official documents of Consiglio Nazionale delle Ricerche [30,31,32].

These diverse longitudinal data allowed a triangulation from multiple sources to explain dynamics of scientific production of the CNR [33]. In fact, the use of secondary data, as considered in the study here, plays more and more a vital role for scientific research of public and private research organizations [34]. The study design also constructs bar graphs of critical scientific activities, and trends of scientific productivity and total revenue per researchers over time. These trends are analyzed with a linear model for scientific productivity and quadratic model for total revenue, both as function of time (1999–2019 period). The specification of models is:

(1) Linear model: Scientific productivity = α+ β *time* + *ε*

(2) Quadratic model: Total revenue = α’ + β_1_*time* − β_2_*time*^*2*^ + *u*

α and α’ = constants; β, β_1,_ β_2_ = coefficients of regression; ε and *u* = error term

Ordinary Least Squares (OLS) method is applied for estimating the unknown parameters of these models (Eq. 1) and (Eq. 2) for a comparative analysis. Statistical analyses are performed with the Statistics Software SPSS version 26.

## 4 Results

The CNR in 2020 had over 8,400 employees and 102 research institutes (associated with more than 330 secondary units and laboratories, including scientific bases in Arctic and Antarctic areas). The research units generate scientific research in manifold fields of research, including natural, engineering and information sciences plus social and human sciences [30,31]. The CNR manages a yearly budget of approximately 1 billion EUR [35]. Public funding for research in CNR is mainly used for the cost of personnel, which has a growth rate higher than revenue (state subsidy and public contracts) over time [36,37]. Since 2010, the research output of Italy's research institutions is evaluated every four years by Italy's research evaluation agency (ANVUR) in the context of the Research Quality Evaluation (VQR) using a peer review approach which, for the “hard science” disciplines (mathematics and computer sciences; physics; chemistry; earth sciences; biology; medicine; agriculture and veterinary sciences; industrial and information engineering; civil engineering and architecture) affords results consistent with bibliometric techniques [38].

The last VQR assessment of the research output of the CNR from 2011 to 2014 showed that the institution under study here was 1^st^ amid Italy's public research bodies evaluated in earth sciences, 7^th^ in physics, 2^nd^ in medical sciences, 4^th^ in life sciences, and 3^rd^ in chemical sciences [39]. Several scientometric studies have investigated Italy's university system [38,40,41] and reorganization of the CNR, which clearly showed a shift from basic to applied research and consultancy to firms offered by new institutes that are more and more operating as quasi-business firms [11].

### 4.1 Typologies and evolution of scientific activities at CNR, 2000–2019 period

Researchers of Italy's CNR, from 2000 to 2019, published 135,262 documents, 72% of which were scientific articles reporting original findings, 18% conference papers, and 4.6% reviews (Table 1).

**Table 1:** Document type and number of published documents by CNR scholars from 2000 to 2019 (Source: Scopus, 2020).

Rank	Document type	No. of documents
1	Article	97,152
2	Conference Paper	24,487
3	Review	6,221
4	Book Chapter	3,280
5	Editorial	1,484
6	Letter	885
7	Note	558
8	Erratum	557
9	Short Survey	320
10	Book	218

Showing evidence of their contribution to scientific education, the CNR scholars authored or co-authored 218 scientific books in English (one of which was published in open access): 44 books were in human sciences (31 in social sciences and 13 in business, management and accounting). The subject areas of research published by CNR scholars, from 2000 to 2019, is led by chemical sciences (chemistry + chemical engineering), followed by physical (physics and astronomy) and life sciences (Table 2).

**Table 2:** Ranking of documents published by CNR scholars per subject area from 2000 to 2019 (Source: Scopus, 2020).

Rank	Subject area	Number of documents 2019
1	Chemical sciences (Chemistry + Chemical Engineering)	33,221 (24,171 + 9,050)
2	Physical sciences (physics and astronomy)	31,974
3	Life sciences (Biochemistry, Genetics and Molecular biology + Immunology and Microbiology)	30,008 (26,127 + 3,881)
4	Engineering	23,433
5	Materials Science	23,233
6	Medicine	17,489
7	Computer Science	16,529
8	Earth and Planetary Sciences	14,449
9	Agricultural and Biological Sciences	14,193
10	Mathematics	10,284
11	Environmental Science	9,221
12	Pharmacology, Toxicology and Pharmaceutics	5,366
13	Neuroscience	4,840
14	Energy	3,825
15	Social Sciences	3,465
	Other	10,565

Scientific research at the CNR in the 21^st^ first two decades is highly collaborative on international scale in line with patterns of international scientific collaboration [42,43]. The international dimension of scientific articles from researchers of Italy's CNR, in the 2000–2019 period, is due to co-authorship with scholars of more than 150 countries, including Malawi, Rwanda, Faroe Islands, Macao and Suriname.

The first scientific journal into the list of top ten journals and conference proceedings with more contributions from CNR scholars during 2000–2019 period is a computer science journal series publishing proceedings, post-proceedings, and monographs (i.e., *Lecture Notes in Computer Science*). The last journal in the list is *Acta Horticulturae* (Table 3).

**Table 3:** Top 10 journals hosting contributions from CNR scholars during 2000–2019 period (Source: Scopus, 2020).

Rank	Journals	No. of documents	Impact Factor (SciScore) in 2019
1	*Lecture Notes In Computer Science*	1,994	1.17
2	*Proceedings of SPIE - The International Society For Optical Engineering*	1,775	0.56
3	*Physical Review B*	1,172	3.736
4	*PLOS One*	1,128	2.776
5	*Scientific Reports*	993	4.011
6	*Optics Infobase Conference Papers*	952	(0.02)
7	*Physical Review Letters*	807	9.227
8	*Applied Physics Letters*	798	3.521
9	*AIP Conference Proceedings*	639	-
10	*Acta Horticulturae*	630	0.230

Alone, the top 10 journals published 10,888 documents, namely the 8 per cent of the overall scientific production of the CNR in the last two decades, suggesting a high concentration of scientific activities in specific research fields [44,45]. In addition, physicists at CNR concentrate publications in old reputed journals, namely three journals (*Physical Review B, Applied Physics Letters*, and *Physical Review Letters*) out of ten in the list are devoted to physics only [44,45].

Only two journals in the list have impact factor above 4.0 points, whereas two journals are published in Open Access (OA) format only (*Scientific Reports* and *PLOS One*) showing early penetration of the open science culture amid CNR scholars. Scholars of chemistry at CNR distribute their publications amid tens of different journals; the list does not include a single chemistry or chemical engineering journal (Table 3).

### 4.2 Scientific production over 2000–2019

The comparison of the scientific production by CNR scholars in 2000 and 2019 reveals important findings. In the year 2000, scholars of CNR institutes published 3,984 documents. The scholarly output increased to 9,423 publications in 2019 (Table 4).

**Table 4:** Document type published by CNR scholars in 2000 and 2019 with percent increment (Δ) % (Source: Scopus, 2020).

Rank	Document type	No. of documents in 2019	No. of documents in 2000	Δ% 2000–2019
1	Article	6,748	3,330	102.64
2	Conference Paper	1,481	497	197.99
3	Review	617	90	585.56
4	Book Chapter	203	6	3283.33
5	Editorial	151	14	978.57
6	Erratum	76	5	1420.00
7	Letter	59	22	168.18
8	Note	55	7	685.71
9	Short Survey	20	9	122.22
10	Data Paper	13	4 (Abstract report)	225.00

**Figure 1:**
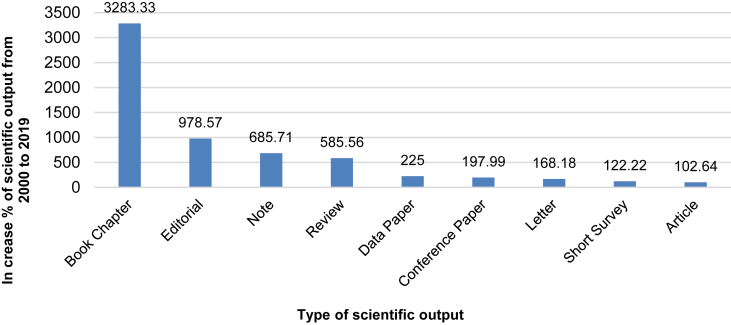
Increase (%) of scientific output from Italy's Research Council, 2000–2019 (Source: Scopus, 2020).

Figure 1 shows the increase percent of different typologies of scientific documents at CNR over 2000–2019 period. In 2000, 85% of documents were scientific articles in peer-reviewed journals. Reflecting the largely increased number of scientific conferences and new communication channels of the digital era including book chapters, the latter percentage decreased to 67% twenty years later.

The comparison between subject areas and related number of scientific contributions at the beginning (in 2000) and at the end of period under study (in 2019) shows evidence of changes in the scientific research carried out by CNR scholars, regardless of several former CNR institutes having entered the new astrophysics (INAF) and earth sciences (INGV) national institutes. The top three disciplines remained the same, namely chemical, life, and physical sciences (Table 5).

**Table 5:** Subject area and number of documents published by CNR scholars in 2000 and in 2019 (Source: Scopus, 2020).

Rank	Subject area	No. of documents in 2000	No. of documents in 2019
1	Chemical sciences (Chemistry + Chemical Engineering)	1,445 (882 + 278)	2,219 (1,504 + 715)
2	Life Sciences (Biochemistry, Genetics and Molecular Biology + Immunology and Microbiology)	1,062 (943 + 119)	1,991 (1,702 + 289)
3	Physical sciences (physics and astronomy)	1,030	1,797
4	Engineering	561	1,787
5	Materials Science	696	1,619
6	Computer Science	350	1,450
7	Medicine	497	1,409
8	Agricultural and Biological Sciences	396	1,142
9	Earth and Planetary Sciences	489	1,003
10	Environmental Science	178	858
11	Mathematics	251	749
12	Energy	47	421
13	Social Sciences	60	375
14	Pharmacology, Toxicology and Pharmaceutics	191	368
15	Neuroscience	153	326
16	Arts and Humanities	61	144

However, the growth of contributions in energy (+896%) and environmental science (+493%) largely outperformed the general growth for all disciplines (250%). Enhanced growth rates are also observed for engineering (+318%), mathematics (+298%), and agricultural and biological sciences (288%, cf., Table 5).

Increase of contributions into the life sciences was +187%, followed by +174% for physical sciences (physics and astronomy). This dynamics shows the shift from basic to applied science: growth in chemical sciences was +153% but, pointing to the enhanced role of applied research, growth in chemical engineering was +257%, in line with the observed convergence between applied and basic research fields generated in the last four decades in dynamics of international scientific collaboration [43].

The comparison between top 10 journals selected by CNR scholars to publish the outcomes of their scientific research in 2000 and in 2019 shows several significant changes (Table 6).

**Table 6:** Top 10 journals and Impact Factor (IF) hosting contributions from CNR scholars in 2000 and in 2019 (Source: Scopus, 2020).

Rank	Journal (Journal Impact Factor) in 2000	No. of documents	Journal (Journal Impact Factor) in 2019	No. of documents
1	*Astronomy and Astrophysics* (5.636)	58	*Scientific Reports* (4.011)	180
2	*Proceedings of SPIE - The International Society for Optical Engineering* (0.36)	54	*Lecture Notes in Computer Science* (1.17)	127
3	*Lecture Notes in Computer Science* (1.17)	46	*Optics Infobase Conference Papers* (No IF)	116
4	*Acta Horticulturae* (0.230)	45	*International Journal of Molecular Sciences* (4.556)	114
5	*Astrophysical Journal* (5.745)	35	*Proceedings of SPIE - The International Society for Optical Engineering* (0.56)	81
6	*European Journal of Organic Chemistry* (2.889)	29	*Science of the Total Environment* (6.551)	72
7	*Tetrahedron Letters* (2.379)	24	*Ceur Workshop Proceedings* (No IF)	68
8	*Inorganica Chimica Acta* (2.304)	23	*Molecules* (3.267)	49
9	*Journal of Cultural Heritage* (1.111)	22	*Remote Sensing* (4.509)	48
10	*Materials Research Society Symposium Proceedings* (No IF)	22	*Journal of Physical Chemistry C* (4.189)	23

The rank in 2000 was based on a specialized physics journal (i.e., *Astronomy and Astrophysics*), whereas in 2019 the new rank started with an OA journal of general science (*Scientific Reports*). More in detail, out of 9,423 documents published by CNR scholars in 2019, 3,228 (i.e., 34%) were freely accessible according to the OA publishing model, and thus to the emerging open science [46], whereas 66% was published in journals for subscribers and pay books. The percentage of OA documents in 2000 was limited to a mere 8.4% (only 337 documents).

Beyond *Scientific Reports*, the 2019 ranking includes three more OA journals, all of which are specialized in chemical sciences (*International Journal of Molecular Sciences*, *Molecules* and *Remote Sensing*). It is also relevant that the 2019 ranking includes a multi-disciplinary journal in environmental science (*Science of the Total Environment*) publishing research on environment, which interfaces the atmosphere, lithosphere, hydrosphere, and biosphere. Finally, from 2000 to 2019 period, the number of journals publishing proceedings amid the top 10 journal platforms selected by CNR scholars went from 3 in 2000 to 4 in the year 2019. On one hand, in 2019 the Journal Impact Factor (JIF) of the proceedings journals was low or even absent. On the other hand, in 2019 the JIF of the journals amid the top 10 selected by CNR scholars was, of course, significantly higher than 2000.

A main finding of the scientific research carried out at the CNR is its increasing collaborative nature within international networks [42]. In 2019, CNR scholars published scientific documents with co-authors of 119 countries, including the Vatican City State (an astrophysics study co-authored with a scholar based at the Specola Vaticana) [47]. Twenty years before, the number of countries having collaborating research with CNR was 7.

**Figure 2:**
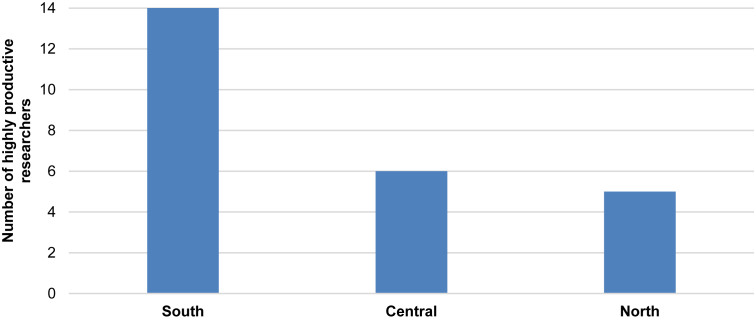
Number of highly productive researchers at CNR per geographic macro areas. (Source: Scopus, 2020).

**Figure 3:**
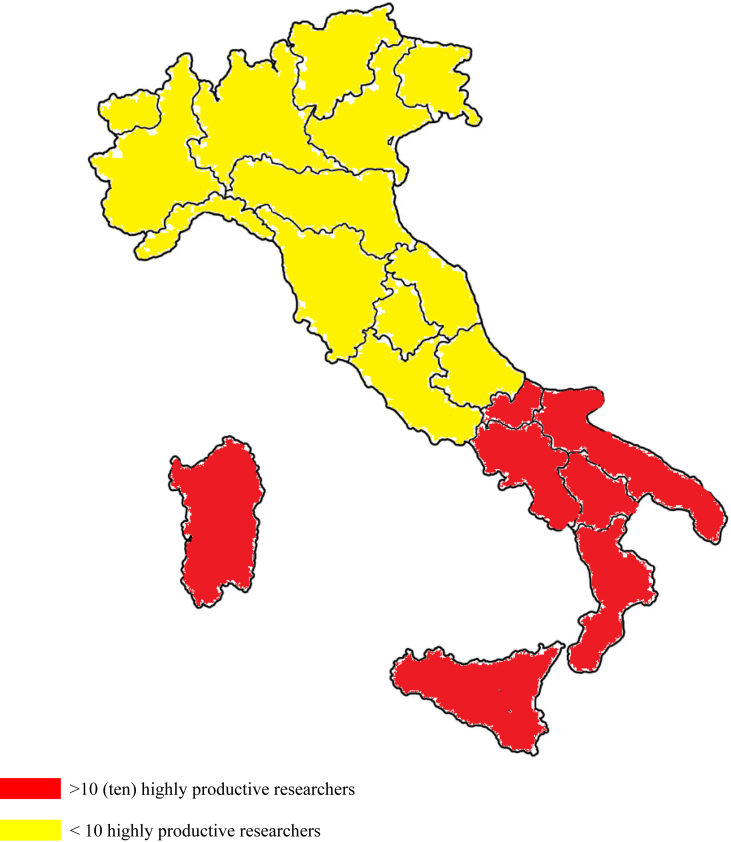
Map of Italy's Research Council with highly productive researchers. (Source: Scopus, 2020).

Finally, Figure 2 and Figure 3 show the geographical location of top productive researchers at CNR: the top 25 prolific scholars over 2000–2019 period are chiefly working in labs of South Italy, such as in regions of Campania (7), Puglia (2), Sicilia (2), Calabria (2), and Basilicata (1). Toscana, in the central part of Italy with 5 scholars, is the second region hosting the most prolific CNR scholars, Veneto region in North Italy (3 scholars) is third. Regions of Umbria (1), Lombardia (1) and Trentino Alto Adige (1) each host one highly prolific scholar. The underlying mechanism of these prolific scholars can be due to the determinant of self-determination in national and international competitive contexts of scientific research [48].

**Figure 4:**
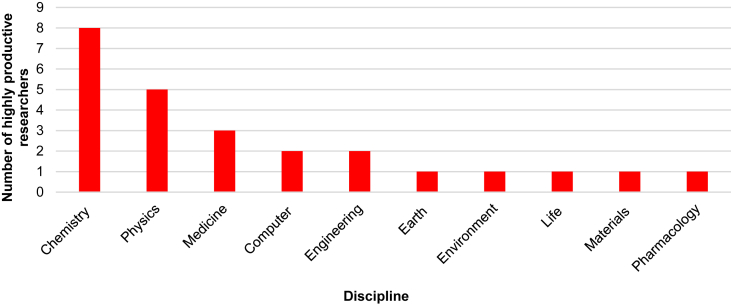
Highly productive researchers of the CNR per scientific discipline (Source: Scopus, 2020).

Figure 4 shows the research field of highly productive researchers: eight out of 25 CNR top scholars work in chemistry, five in physics, three prolific scholars perform research in medicine, two in computer science and in engineering, and one each in pharmacology, materials, life, earth and environmental sciences.

### 4.3 The relation between scientific productivity and economic resources at CNR over time and the driver of self-determination of scholars

**Figure 5:**
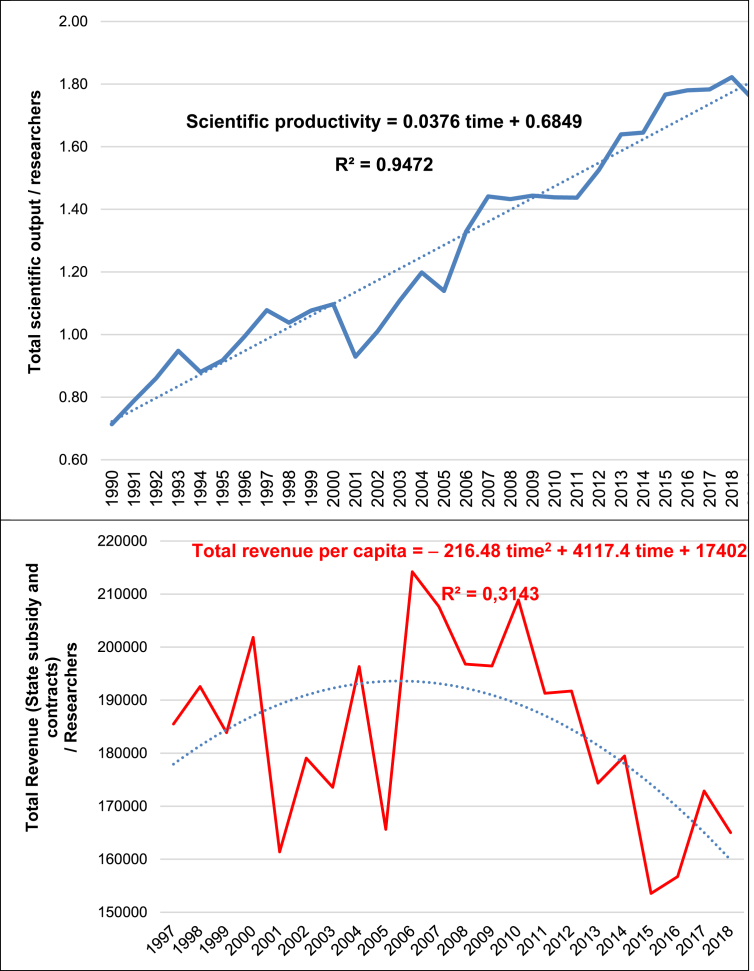
Scientific productivity (*top*) and revenue (*bottom*) of researchers at Italy's CNR, 1990–2019 period (Source: Scopus, 2020).

Figure 5 (top) shows that the scientific productivity of researchers at CNR is increasing over time, whereas the level of state subsidy and contracts per researcher is reducing over time (Figure 5, bottom). This comparative analysis suggests the driving role of scholars in supporting scientific productivity of CNR (based on a higher quantity and quality of scientific production at international level, as described before), though less and less economic resources because of shrinking public research lab budgets and in general of economic resources. Estimated relationships with OLS methods quantify these trends of Figure 5 with a high goodness of fit:

Linear model, Scientific productivity = 0.685 + 0.038 *time* + ε (R^2^ = 95%)

Quadratic model, Total revenue = 174026 + 4117.4 *time* − 216.48 *time*^*2*^ + *u* (R^2^ = 31.4%)

This finding raises a main question about the drivers of this high performance of scholars in the presence of limited resources and continuous organizational reforms that have created instability in the organizational behavior of Italian institutes within CNR. This result can be explained with the vital factor of self-determination of scholars that seems to be reinforced in problematic situation of crises.

In general, human nature has the natural tendency to seek out novelty and challenges, to extend and exercise one's capacities, to explore, and to learn. The self-determination here is driven by intrinsic motivation of scholars and human tendency towards learning and creativity that support motivation, performance, and well-being of people in organizations and society [49]. As a matter of fact, intrinsic motivation exists in the scientific research itself and gives personal satisfaction to scholars, such as autonomy, recognition, trust, and empowerment [50,51]. Autonomy in science is not only to being independent, detached, or selfish but rather it is associated with the feeling of volition that can accompany any act, whether dependent or independent, collectivist or individualist.

The self-determination is due to satisfaction of scholars in performing scientific activity and to increase their reputation in scientific communities [48,52]. The theory suggests that scholars are intrinsically motivated if they perform research tasks based on an internalized sense of duty and/or enjoyment also with the goal of career advancements and higher reputation in science. Autonomy and competence support intrinsic motivation of scholars in association with a third vital factor that is relatedness. These three elements support personality development and behavioral self-regulation of scholars, improving personal wellbeing and scientific performance of organizations, though reduction of funding as in Italy [53,54].

## 5 Discussion and R&D management implications

Current organization of research bodies, designed to stimulate technology transfer, technological services and the commercialization of research, is in line with the so-called “academic capitalism” [55], characteristic also of today's universities [56]. However, research units of CNR observe that there is an unfulfilled (and perhaps even unfulfillable) role of departments in the acquisition of large projects, and they are called upon to secure third party contracts (of various kinds) to compensate for the decreasing governmental funds. This compensation can assure the scientific quality in the long run [11,16,17,18,19].

This strategic reorientation of research units, more and more market-oriented, can be seen as a functional adaptation to a growing culture of retrenchment. As a consequence, public research units adopt a market orientation similar to “quasi-business firms, with many characteristics of business firm, except for the profit motive” [57]; but they also generate higher levels of bureaucratization to manage their new activities, which are based mainly on a mix of small and medium research projects, and consulting activities for external clients [13,17].

According to Greenfield, such “embracement of the market is compromising scientific norms and commercialization (or commodification, or marketization) is in profound conflict with the function and main mission of research units and universities” [58], though being the result -- the transfer of norms and practices from the industrial to the academic sector -- of the industrialization of higher education and research [59,60]; moreover, “unless [marketization of the scientific research] is halted soon, important portions of future scientific knowledge will be private property and fall outside the public domain” [61]. Goldfarb [62] argues that researchers who maintain a relationship with the sponsor of their research “experience a decrease in publications in leading international journals, implying [the danger] that academics’ careers may become a function of their funding rather than their talent” [62].

In Italy, the lack of a long-term national research strategy and the absence of a consistent research policy shared by various governmental coalitions weakens the national system of innovation, and strains the overall economy [11]. The hastily implemented and erratic Italian public management reforms have created problems within research organization, particularly due to a “massification” -- and consequential dilution -- of scientific research. However, self-determination of scholars in this new situation is a critical driver supporting more and more scientific production and productivity in terms of high quantity and quality of scientific outputs with increasing international collaborations in networks of science.

### 5.1 Theoretical contribution: self-determination as driver of scientific production

This study reveals that self-determination of scholars, especially of specific geographical areas, is a main driver of scientific performance also in the presence of scarce economic resources. In particular, in science sector, self-determination is driven by vital factors of intrinsic motivation in science, such as autonomy, competence, and relatedness that support personality development and international reputation of scholars in scientific communities, organizations and society [63]. These factors seem to be reinforced in problematic situations associated with lack of financial resources and crises, when scholars direct all scientific competencies to solve overriding problems in society, such as in the presence of Coronavirus disease 2019 when the scientific production in this new field of research has grown at accelerated rates of production in Italy.

### 5.2 R&D management implications: the vital role of intrinsic incentive in science

Results of this study suggest that R&D management of labs has to support intrinsic incentives that have a powerful effect on performance, motivation, commitment, and satisfaction of scholars in organizations [64,65]. Lincoln and Kalleberg argue that incentives may have a powerful effect on employees’ attitudes and motivations towards their job and the organization for which they work [66]. In the context of incentive management, this study suggests that R&D management of public research organizations should also focus on goal theory to design best practices to drive motivation and intrinsic incentives of scholars for supporting individual scientific productivity and as a consequence overall organizational performance.

This managerial theory suggests that people's goals play an important role in determining behavior and performance of organization. In short, challenging goals can reinforce scientific behavior and performance of scholars and organizations. Moreover, scholars having difficult goals in the presence of scarce resources will perform better than people with easier goals. In short, the theory of goal provides a useful approach for R&D management to improve motivation, incentive, and scientific performance in public research organizations [67]. Overall, then, best practices of goal theory provide fruitful R&D managerial implications considering incentive systems, such as: specific performance goals should systematically be identified and set in order to direct behavior and maintain high motivation of scholars; incentives should be given by setting scientific goals at a challenging but realistic level, since difficult scientific goals lead to higher motivation and performance of scholars; finally, scholars' participation in the setting of goals is a main intrinsic incentive that may lead to higher motivation, satisfaction and work involvement that increase performance in scientific organizations [50,54].

## 6 Concluding remarks and limitations

Six main findings emerge from case study of the Italy's National Research Council from 2000 to 2019.

*First*, the yearly scientific output from 2000 to 2019 is almost tripled (+250%) at a significantly higher rate than number of researchers and technologists, which went from 3,625 in 2000 to 5,418 in 2019 (+150%).

*Second*, aware of the importance of open science, CNR scholars in 2019 published a relatively high percentage (34%) of their research results in OA journals, supporting worldwide diffusion of studies [46].

*Third*, three main fields of research at the CNR -- chemical, life, and physical sciences -- remained the same regardless of significant increases in the volume of research devoted to energy, environmental science, engineering, mathematics, and agricultural science. This result confirms the high tradition and path-dependence of the CNR in supporting scientific advances in natural and engineering science.

*Fourth*, research at Italy's CNR was already highly internationalized in 2000, with collaborations in 77 countries; in 2019 scientific activity is completely internationalized with increasing collaborations with abroad scholars of more than 119 countries.

*Fifth*, the top prolific scholars of the CNR (14 out 25) are mainly based in Italy's southern regions, all of which are substantially less economically developed areas and where the research performance (in the “hard” sciences) of university professors in South Italy is on average lower than professors in North Italy [68]. This result reveals the highest potential of human resources at CNR, having excellent scientific and technical skills in some research institutes headquartered in Italy's southern regions and other parts of Italy.

*Finally*, the study here also reveals an increasing scientific productivity of CNR researchers driven by self-determination mechanisms [49], though shrinking public research lab budgets and some organizational and managerial deficiencies [11].

To conclude, although this analysis is focused on a case study of Italy, and the findings cannot be simply transferred to other research organizations, the worldwide trend in research sector seems to run in parallel in many countries. This aspect is due to universities and public research bodies that exhibit a similar organizational behavior in a globalized world [69]. Such forebodings are relevant to modern, knowledge-driven economies in their support strategies for sensible changes regarding public research units and universities [70].

Overall, then, policy-makers and R&D mangers have to be aware of vital factor of intrinsic motivation of scholars to design appropriate best practices that can improve the satisfaction and involvement of personnel in scientific tasks and as a consequence support efficiency of organizational behavior and higher performance of research institutions also in the presence of scarce funds. In this manner, public managers and policy-makers can improve the organizational behavior of public research institutions that play a driving role as “engines of growth,” based on their intangible capital, brainpower [71]. Of course, further and extended researches concerning the organizational and managerial behavior of other public research institutions and universities are needed, a kind of meta-research, and new ways have to be found to foster, not hinder, the relevant scientific activities of research institutions in current society with rapid changes and unforeseen crises.

## Declarations

### Author contribution statement

All authors listed have significantly contributed to the investigation, development and writing of this article.

### Funding statement

This research did not receive any specific grant from funding agencies in the public, commercial, or not-for-profit sectors.

### Data availability statement

Data included in article/supplementary material/referenced in article.

### Declaartion of interests statement

The authors declare no conflict of interest.

### Additional information

No additional information is available for this paper.

## References

[1] Coccia M. (2019). Why do nations produce science advances and new technology?. Technol. Soc..

[2] Coccia M. (2001). New models for measuring the R&D performance and identifying the productivity of public research institutes. R D Manag..

[3] Coccia M. (2004). A basic model for evaluating R&D performance: theory and application in Italy. R D Manag..

[4] Coccia M. (2005). A scientometric model for the assessment of scientific research performance within public institutes. Scientometrics.

[5] Coccia M. (2008). Measuring scientific performance of public research units for strategic change. J. Informetr..

[6] Gioia D., Chittipeddi K. (1991). Sensemaking and sensegiving in strategic change initiation. Strat. Manag. J..

[7] Sanz-Menéndez L., Van Ryzin G.G. (2015). Economic crisis and public attitudes toward science: a study of regional differences in Spain. Publ. Understand. Sci..

[8] Cruz-Castro L., Sanz-Menéndez L. (2018). Autonomy and authority in public research organisations: structure and funding factors. Minerva.

[9] Cruz-Castro L., Sanz-Menéndez L. (2016). The effects of the economic crisis on public research: Spanish budgetary policies and research organizations. Technol. Forecast. Soc. Change.

[10] Coccia M. (2005). A taxonomy of public research bodies: a systemic approach. Prometheus.

[11] Coccia M. (2008). New organisational behaviour of public research institutions. Int. J. Bus. Innovat. Res..

[12] Coccia M., Rolfo S. (2007). How research policy changes can affect organization and productivity of public research institutes? Analysis within the Italian national system of innovation. J. Comp. Pol. Anal..

[13] Coccia M. (2009). Bureaucratization in public research institution. Minerva.

[14] Coccia M. (2009). Research performance and bureaucratization within public research labs. Scientometrics.

[15] Carayol N., Matt M. (2004). Does research organization influence academic production? Laboratory level evidence from a large European university’. Resour. Pol..

[16] Coccia M., Rolfo S. (2002). Technology transfer analysis in the Italian national research council. Technovation.

[17] Coccia M., Rolfo S. (2009). Project management in public research organization: strategic change in complex scenarios. Int. J. Proj. Organisat. Manag..

[18] Coccia M., Rolfo S. (2010). New entrepreneurial behaviour of public research organizations: opportunities and threats of technological services supply. Int. J. Serv. Technol. Manag..

[19] Coccia M., Rolfo S. (2008). Strategic change of public research units in their scientific activity. Technovation.

[20] Mangematin V., Blanco S., Deschamp B., Genet C., Kahane B. (2006). Project Management: Learning by Breaking the Rule, *Working Papers 2006-06*.

[21] Sydow J., Lindkvist L., De Fillippi R. (2004). Project-based organizations, embeddedness and repositories of knowledge. Organ. Stud..

[22] Coccia M., Falavigna G., Manello A. (2015). The impact of hybrid public and market-oriented financing mechanisms on scientific portfolio and performances of public research labs: a scientometric analysis. Scientometrics.

[23] Zeller C. (2002). Project teams as means of restructuring research and development in the pharmaceutical industry. Reg. Stud..

[24] Miller R., Lessard D. (2001). Understanding and managing risks in large engineering projects. Int. J. Proj. Manag..

[25] Mihm J., Loch C., Huchzermeier A. (2003). Problem-solving oscillations in complex engineering projects. Manag. Sci..

[26] Eisenhardt K.M. (1989). Building theories from case study research. Acad. Manag. Rev..

[27] Eisenhardt K.M., Graebner M.E. (2007). Theory building from cases: opportunities and challenges. Acad. Manag. Rev..

[28] Ansari S.S., Garud R., Kumaraswamy A. (2016). The disruptor’s dilemma: TiVo and the U.S. Television ecosystem. Strat. Manag. J..

[29] (September 8, 2020). Queries Carried Out Online on scopus.Com Using Boolean Operators.

[30] Consiglio Nazionale delle Ricerche (2020). Il Cnr in Numeri. https://www.cnr.it/it/cnr-in-numeri.

[31] Consiglio Nazionale delle Ricerche, Statistiche C.N.R. (2020). Personale in Servizio. http://www.dcp.cnr.it/DPUASI/statwork.asp.

[32] Consiglio Nazionale delle Ricerche (2020). Documenti di bilancio, 1999-2019. https://www.cnr.it/it/documenti-bilancio.

[33] Jick T.D. (1979). Mixing qualitative and quantitative methods: triangulation in action. Adm. Sci. Q..

[34] Kozinets R.V. (2002). The field behind the screen: using ethnography for marketing research in online communities. J. Mar. Res..

[35] Consiglio Nazionale delle Ricerche (2020). Rendiconto Generale Per L'esercizio Finanziario 2019, Rome. http://www.cnr.it/sites/default/files/public/media/bilancio_bk/rendiconto_generale_2019_RF2019_dgt.pdf.

[36] Coccia M. (2019). Metabolism of public organizations: a case study. J. Soc. Adm. Sci..

[37] Coccia M. (2014). Structure and organisational behaviour of public research institutions under unstable growth of human resources. Int. J. Serv. Technol. Manag..

[38] Abramo G., D’Angelo C.A., Caprasecca A. (2009). Allocative efficiency in public research funding: can bibliometrics help?. Resour. Pol..

[39] ANVUR, Valutazione della Qualità della (2017). Ricerca 20112014 - (VQR 2011-2014) - L’analisi Delle Singole Strutture: Il Consiglio Nazionale Delle Ricerche (CNR), Rome. https://www.anvur.it/rapporto-2016/files/Enti/99.CNR.pdf.

[40] Peroni S., Ciancarini P., Gangemi A., Nuzzolese A.G., Poggi F., Presutti V. (2020). The practice of self-citations: a longitudinal study. Scientometrics.

[41] Abramo G., D’Angelo C.A., Soldatenkova A. (2017). How long do top scientists maintain their stardom? An analysis by region, gender and discipline: evidence from Italy. Scientometrics.

[42] Coccia M., Bozeman B. (2016). Allometric models to measure and analyze the evolution of international research collaboration. Scientometrics.

[43] Coccia M., Wang L. (2016). Evolution and convergence of the patterns of international scientific collaboration. Proc. Natl. Acad. Sci. U.S.A..

[44] Coccia M. (2018). General properties of the evolution of research fields: a scientometric study of human microbiome, evolutionary robotics and astrobiology. Scientometrics.

[45] Coccia M. (2020). The evolution of scientific disciplines in applied sciences: dynamics and empirical properties of experimental physics. Scientometrics.

[46] Pagliaro M. (2020). Publishing scientific articles in the digital era. Open For. Sci. J..

[47] Bini D., Geralico A., Gionti G., Plastino W., Velandia N. (2019). Scattering of uncharged particles in the field of two extremely charged black holes. Gen. Relat. Gravit..

[48] Deci E.L., Ryan R.M. (1985). Intrinsic Motivation and Self-Determination in Human Behavior.

[49] Baard P.P., Deci E.L., Ryan R.M. (2004). Intrinsic need satisfaction as a motivational basis of performance and well-being at work: an application of cognitive evaluation theory. J. Appl. Soc. Psychol..

[50] Coccia M., Farazmand A. (2019). Comparative incentive systems. Global Encyclopedia of Public Administration, Public Policy, and Governance.

[51] Coccia M., Benati I. (2018). Rewards in public administration: a proposed classification. J. Soc. Adm. Sci..

[52] Deci E.L. (1975). Intrinsic Motivation.

[53] Coccia M. (2018). Motivation and theory of self-determination: some management implications in organizations. J. Econ. Bibliogr..

[54] Coccia M., Farazmand A. (2019). Theories of self-determination. Global Encyclopedia of Public Administration, Public Policy, and Governance.

[55] Slaughter S., Leslie L. (1997). Academic Capitalism: Politics, Policies, and the.

[56] Etzkowitz H. (2003). Research groups as ‘quasi-firm’: the invention of the entrepreneurial university. Resour. Pol..

[57] Viale R., Etzkowitz H. (18-21 May 2005). Third Academic Revolution: Polyvalent Knowledge; the DNA of the Triple helix.

[58] Greenfeld L. (2001). The Spirit of Capitalism: Nationalism and Economic Growth.

[59] C. Musselin, *The Transformation Of Academic Work: Acts And Analysis*, CSHE Research & Occasional Paper Series: CSHE.4.07, **2007**, University of California, Berkeley.

[60] Washburn J. (2005).

[61] Nelson R.R. (2005). The Limits of Market Organization.

[62] Goldfarb B. (2008). The effect of government contracting on academic research: does the source of funding affect scientific output?. Resour. Pol..

[63] Deci E.L., Ryan R.M. (2004). Handbook of Self-Determination Research.

[64] O’Reilly C.A., Caldwell D.F. (1980). Job choice: the impact of intrinsic and extrinsic factors on subsequent satisfaction and commitment. J. Appl. Psychol..

[65] O’Reilly C.A., Chatman J., Caldwell D. (1991). People and organizational culture: a profile comparison approach to assessing person-organization fit. Acad. Manag. J..

[66] Lincoln J.R., Kalleberg A.L. (1990). Culture, Control and Commitment: A Study of Work Organization and Work Attitudes in the United States and Japan.

[67] Locke E.A. (1968). Towards a theory of task motivation and incentives. Organ. Behav. Hum. Decis. Process..

[68] Abramo G., D’Angelo C.A., Rosati F. (2016). The north-south divide in the Italian higher education system. Scientometrics.

[69] Coccia M., Greenfeld L. (2012). Organization and strategic change of public research institutions. The Ideals of Joseph Ben-David.

[70] Bozeman B., Youtie J., Fukumoto E., Parker M. (2019). When is science used in science policy? Examining the importance of scientific and technical information in national research council reports. Rev. Pol. Res..

[71] Corley E.A., Bozeman B., Zhang X., Tsai C.-C. (2019). The expanded scientific and technical human capital model: the addition of a cultural dimension. J. Technol. Tran..

